# Premature Multivessel Coronary Artery Disease in a 31-Year-Old Woman With Ischemic Cardiomyopathy, Left Ventricular Thrombus, Stroke, and End-Stage Renal Disease

**DOI:** 10.7759/cureus.111006

**Published:** 2026-06-17

**Authors:** Sohaib Ahmed, Varun Mehta, Iman Squires, Nelson Reyes Perez, Aliki Gastaud

**Affiliations:** 1 Internal Medicine, University of Central Florida, Orlando, USA; 2 Internal Medicine, HCA Healthcare, Kissimmee, USA

**Keywords:** coronary artery bypass grafting(cabg), end-stage renal disease (esrd), heart failure with reduced ejection fraction, icmp-ischemic cardiomyopathy, left ventricular cardiac thrombus, non-st segment elevation myocardial infarction (nstemi), premature coronary artery disease, stroke

## Abstract

Ischemic cardiomyopathy and multivessel coronary artery disease (CAD) are uncommon in young adults, particularly women younger than 35 years, and are often associated with atypical or aggressive risk factors. The coexistence of severe left ventricular (LV) systolic dysfunction and LV thrombus further increases morbidity because of heightened embolic risk. We present the case of a 31-year-old woman with poorly controlled type 2 diabetes mellitus, hypertension, and chronic kidney disease who presented with dyspnea and edema and was found to have a non-ST-elevation myocardial infarction (NSTEMI). Evaluation revealed severe multivessel CAD, ischemic cardiomyopathy with a left ventricular ejection fraction (LVEF) of 23%, and a large LV apical thrombus on cardiac magnetic resonance imaging. Her hospital course was complicated by acute ischemic stroke, suspected heparin-induced thrombocytopenia, and newly recognized end-stage renal disease (ESRD). Management required complex anticoagulation decisions and delayed surgical revascularization. After prolonged medical optimization, she successfully underwent coronary artery bypass grafting (CABG). This case highlights the diagnostic and therapeutic challenges of advanced ischemic heart disease with LV thrombus in a young woman and underscores the importance of individualized multidisciplinary care in managing rare but high-risk cardiovascular presentations.

## Introduction

Ischemic cardiomyopathy and multivessel coronary artery disease (CAD) have historically been associated with older adults; however, recent epidemiologic data demonstrate an increasing incidence among young and middle-aged individuals. From 1990 to 2021, the age-standardized incidence of ischemic heart disease in persons aged 15-49 years increased by 0.23% annually, while mortality declined by 0.67%. In 2019, the global incidence in this age group was 26.81 per 100,000, with mortality of 7.15 per 100,000. In the United States, approximately 20% of patients hospitalized with myocardial infarction are ≤40 years old [1-2]. Although men continue to have higher overall rates of ischemic heart disease, cardiovascular disease in young women is frequently underrecognized and associated with worse outcomes [3-4].

Young adults with premature CAD commonly possess modifiable cardiovascular risk factors. More than 90% of patients with a first myocardial infarction have at least one major risk factor, including dyslipidemia, diabetes mellitus, hypertension, smoking, obesity, or family history [5]. Severe left ventricular (LV) systolic dysfunction predisposes to LV thrombus formation through the combined effects of endocardial injury and intracardiac stasis. In the contemporary primary percutaneous coronary intervention (PCI) era, LV thrombus occurs in approximately 2.7% of all ST-elevation myocardial infarction (STEMI) cases and 9.1% of anterior STEMI cases, with even higher detection rates when cardiac magnetic resonance imaging (MRI) is utilized [6]. LV thrombus carries a substantial risk for systemic embolization, including ischemic stroke [7].

We present a rare and complex case of a young woman with longstanding diabetes mellitus and uncontrolled hypertension found to have severe multivessel CAD complicated by ischemic cardiomyopathy, LV thrombus, embolic stroke, and dialysis-dependent end-stage renal disease secondary to nodular diabetic glomerulosclerosis.

## Case presentation

A 31-year-old woman with a history of type 2 diabetes mellitus diagnosed at age 14 (hemoglobin A1c 8.6%), uncontrolled hypertension, obesity (body mass index 32 kg/m²), and severe medication nonadherence presented with progressively worsening dyspnea and bilateral lower extremity edema. She reported not taking any prescribed medications before admission.

**Figure 1:**
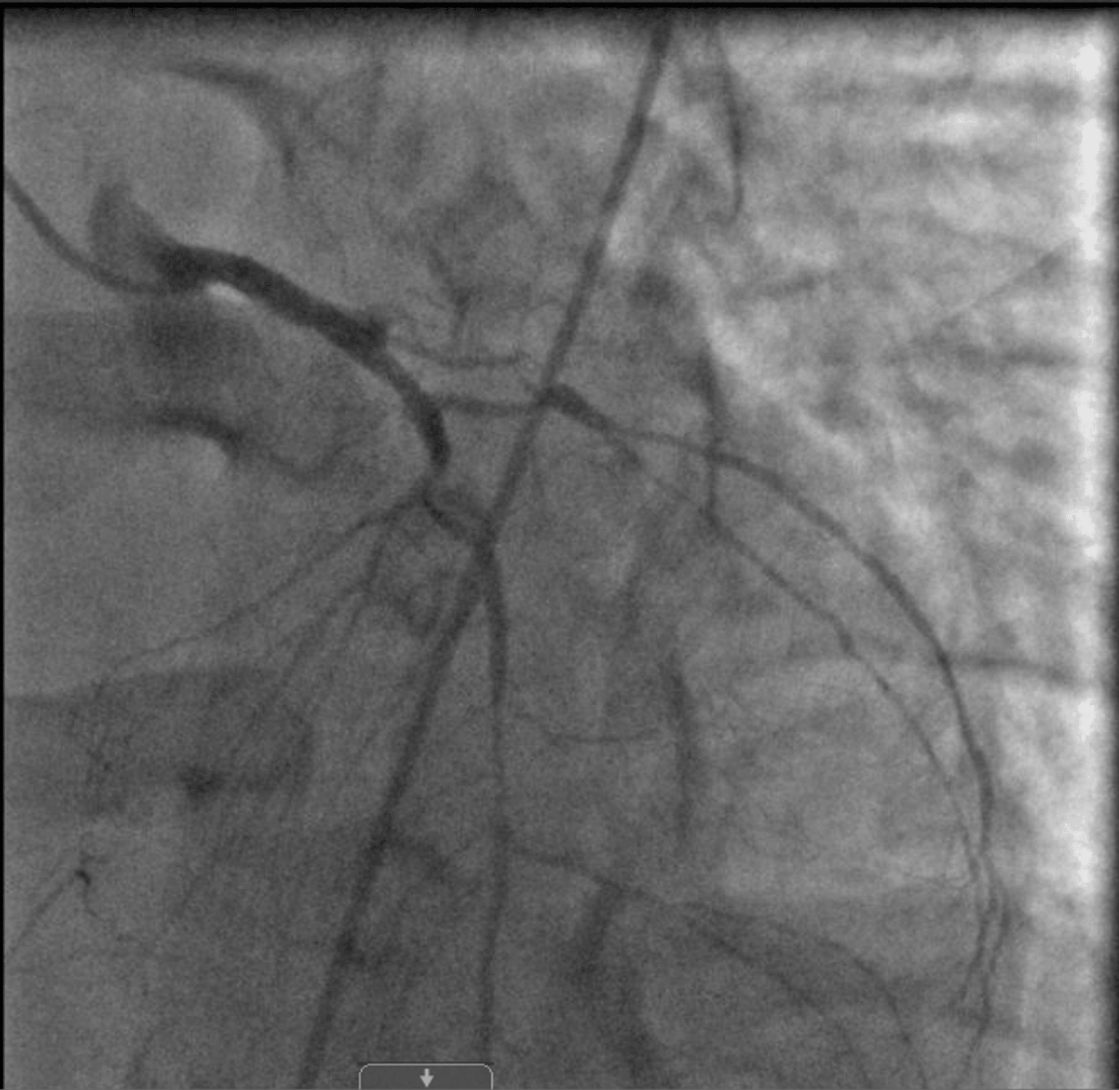
Left anterior oblique cranial view on cardiac catheterization

**Figure 2:**
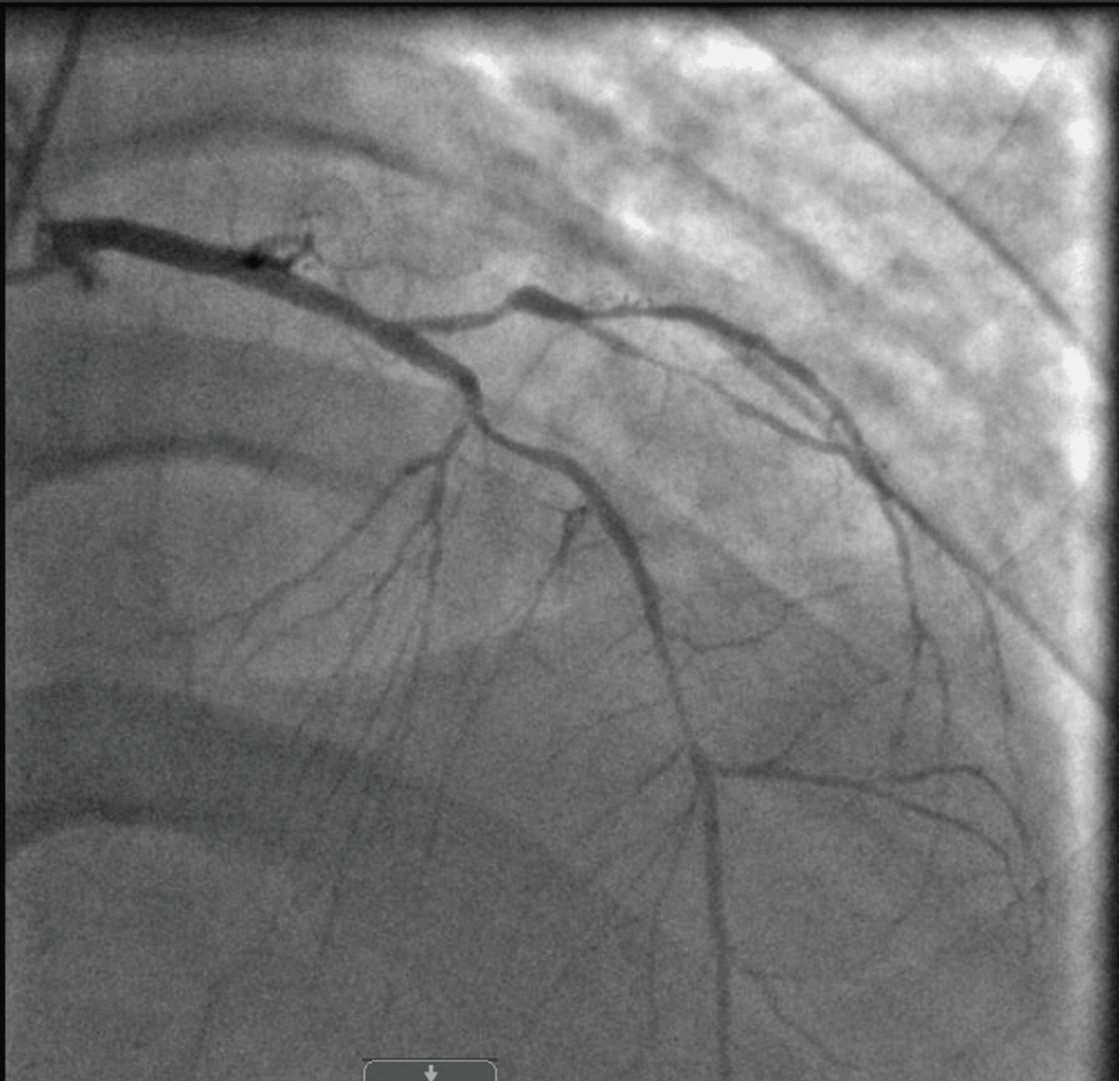
Right anterior oblique caudal view on cardiac catheterization

**Figure 3:**
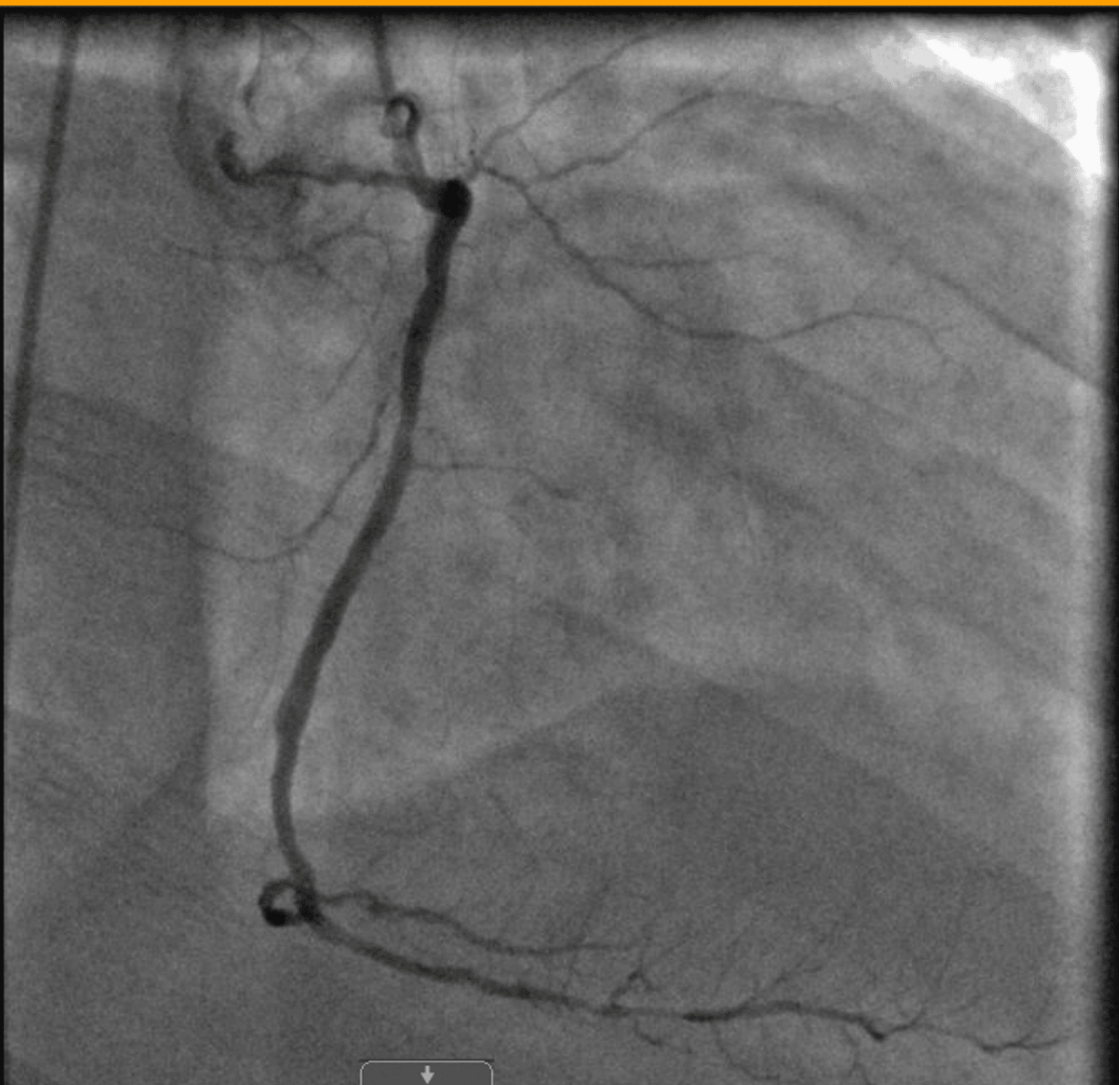
Left anterior oblique view of the right coronary artery on cardiac catheterization

On presentation, she was hypertensive and clinically volume overloaded but remained hemodynamically stable. Laboratory evaluation demonstrated a peak troponin level of 9,291 ng/L, consistent with non-ST-elevation myocardial infarction (Table 1). Coronary angiography revealed severe multivessel coronary artery disease (CAD), including approximately 80% stenosis of the proximal left anterior descending artery and diffuse right coronary artery disease (Figures 1-3). Despite an extensive atherosclerotic burden, her lipid profile was within normal limits with low-density lipoprotein (LDL) cholesterol 85 mg/dL, total cholesterol 146 mg/dL, high-density lipoprotein (HDL) cholesterol 50 mg/dL, and triglycerides 54 mg/dL. Phospholipid antibodies were within the normal range (Table 2). The homocysteine level was elevated.

**Table 1:** Critical labs on presentation BUN: blood urea nitrogen; HbA1C: hemoglobin A1C

Lab Value	Result	Reference Range
BUN	31 mg/dL	7-18 mg/dL
Creatinine	2.91 mg/dL	0.6-1.3 mg/dL
Glucose	184 mg/dL	74-106 mg/dL
High Sensitivity Troponin	9291 ng/L	Female< 54, Male<78 ng/L
HbA1c	8.6%	<5.7%
Heparin-Induced Antibody	1.119 OD	<0.400 OD
Homocysteine	21.41 mcmol/l	5-12 mcmol/l

**Table 2:** Other pertinent labs Ab: antibody; HDL: high-density lipoprotein; LDL: low-density lipoprotein

Lab Value	Result	Reference Range
Serotonin Release Assay	Negative	Negative
Phospholipid IgG Ab	9 units	0-30 units
Phospholipid IgA Ab	1 unit	0-30 units
Phospholipid IgM Ab	15 units	0-30 units
LDL Cholesterol	85 mg/dL	<100 mg/dL
Total Cholesterol	146 mg/dL	<200 mg/dL
HDL Cholesterol	50 mg/dL	>40 mg/dL
Triglycerides	54 mg/dL	<150 mg/dL

**Figure 4:**
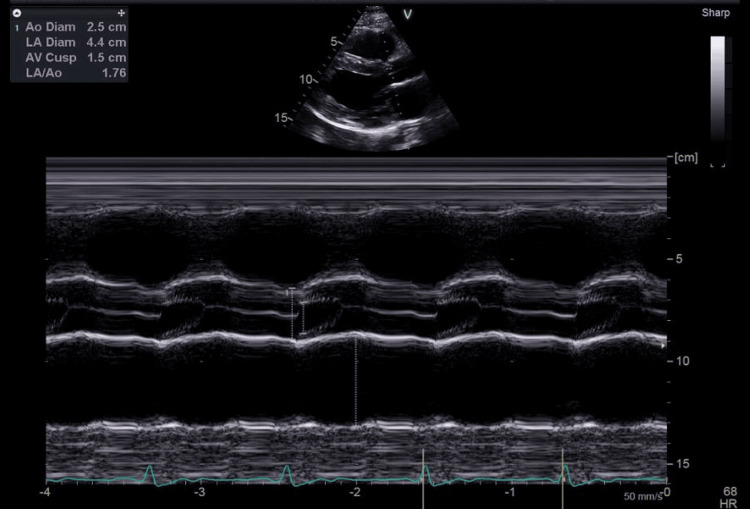
Parasternal long-axis (PLAX) view with M-mode through the left ventricle on echocardiogram

**Figure 5:**
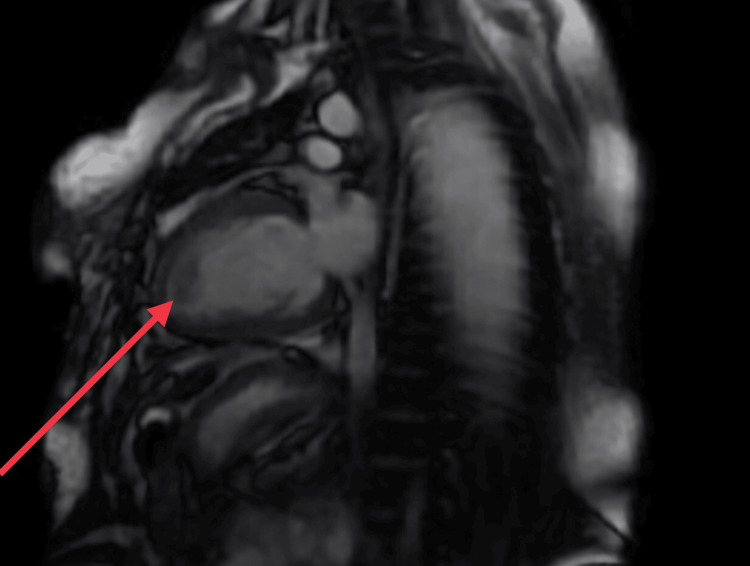
Two-chamber view on cardiac MRI showing apical left ventricular thrombus (arrow)

**Figure 6:**
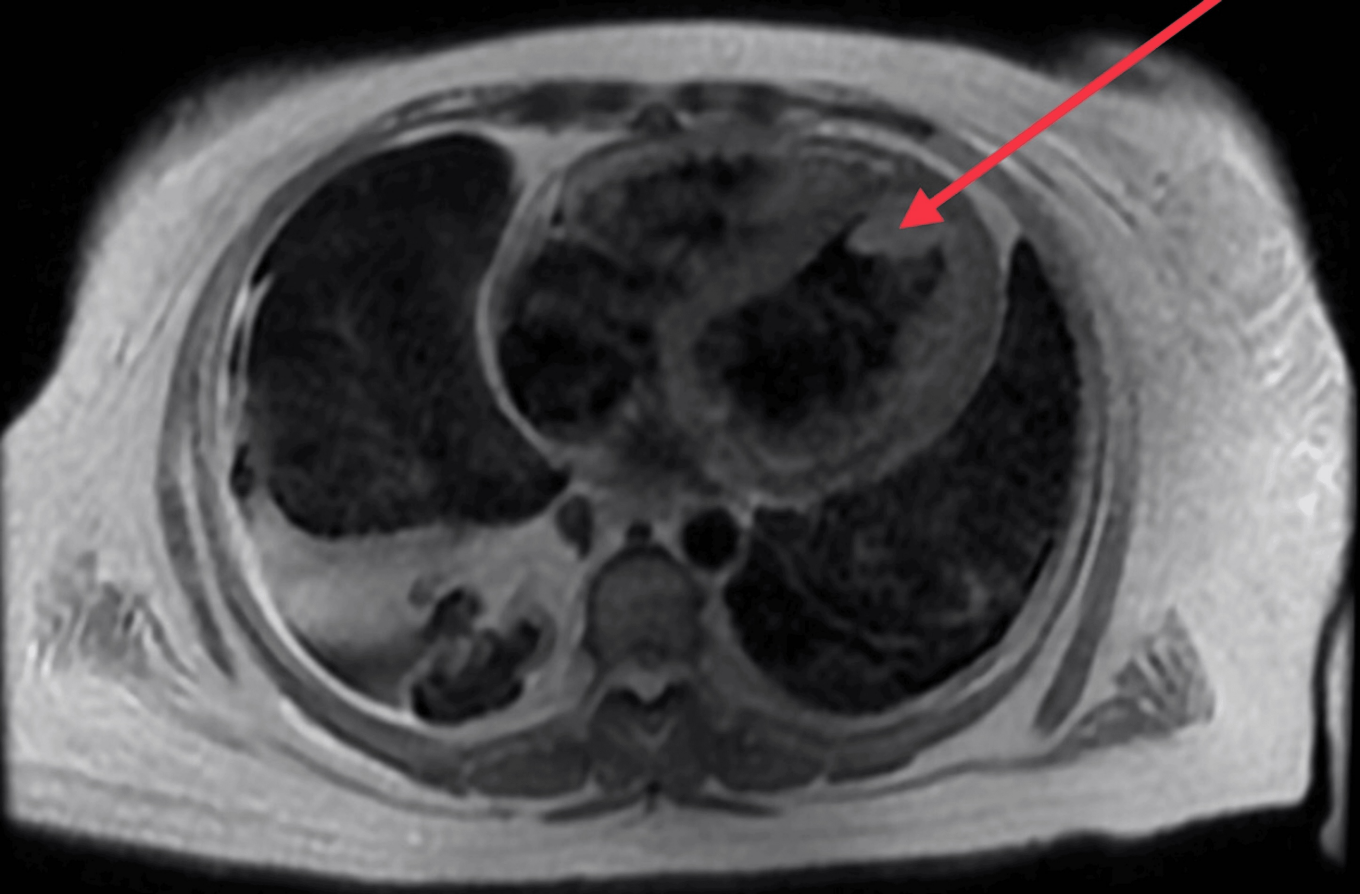
Left ventricular apical thrombus seen on cardiac MRI (arrow)

Transthoracic echocardiography demonstrated moderately reduced LV systolic function with an estimated left ventricular ejection fraction (LVEF) of 35-40% and regional wall motion abnormalities (Figure 4). Subsequent cardiac MRI revealed severe global systolic dysfunction with an LVEF of 23% and identified a 3.1-cm apical LV thrombus (Figures 5, 6).

Therapeutic anticoagulation with unfractionated heparin was initiated. During hospitalization, thrombocytopenia developed in conjunction with positive heparin-platelet factor 4 antibody testing, prompting transition to argatroban for presumed heparin-induced thrombocytopenia. Confirmatory serotonin release assay testing later returned negative. Long-term anticoagulation with warfarin was subsequently continued.

Despite anticoagulation therapy, the patient developed acute visual disturbance and right lower extremity weakness. Brain MRI demonstrated multifocal acute cerebral infarctions consistent with embolic stroke. Thrombolytic therapy was not administered because of recent anticoagulation exposure, uncertain symptom onset timing, and elevated hemorrhagic risk in the setting of severe cardiomyopathy and large LV thrombus.

During the hospitalization, the patient progressed to dialysis-dependent end-stage renal disease secondary to nodular diabetic glomerulosclerosis. Coronary artery bypass grafting (CABG) was initially deferred because of a recent ischemic stroke, severe LV dysfunction, and dialysis dependence. Following seven months of medical optimization and multidisciplinary reassessment, she ultimately underwent successful CABG. Given her diabetes mellitus and multivessel coronary artery disease involving the proximal left anterior descending artery, surgical revascularization with a coronary artery bypass graft (CABG) was deemed the most appropriate management strategy [8]. She was subsequently discharged home in stable condition.

## Discussion

This case illustrates severe premature CAD in a young woman with longstanding metabolic disease and multiple cardiovascular complications. Global epidemiologic data continue to demonstrate a rising incidence of ischemic heart disease among individuals aged 15-49 years despite declining overall mortality. In the United States, approximately one in five myocardial infarctions occurs in patients aged 40 years or younger [1].

Young patients with premature CAD frequently exhibit modifiable cardiovascular risk factors. More than 90% possess at least one major risk factor, most commonly dyslipidemia (90%), diabetes mellitus (41%), hypertension (33-50%), smoking (39%), or obesity (18%) [5]. Our patient demonstrated several high-risk features, including diabetes mellitus beginning in adolescence, poor glycemic control with hemoglobin A1c of 8.6%, uncontrolled hypertension, obesity, and severe medication nonadherence. Although her lipid profile remained within the normal range, diabetes mellitus and hypertension are independently associated with accelerated atherosclerosis and premature CAD [2]. Her presentation is therefore consistent with the increasingly recognized phenotype of severe early-onset atherosclerotic cardiovascular disease.

Of note, elevated homocysteine levels have been epidemiologically associated with an increased risk of cardiovascular disease. Multiple large observational studies and meta-analyses have demonstrated a dose-response relationship between homocysteine concentrations and adverse cardiovascular outcomes. However, randomized trials have shown that lowering homocysteine levels with B-vitamin supplementation does not translate into a reduction in cardiovascular events [9-10]. Our patient was found to have an elevated homocysteine level, which may have represented an additional contributor to her cardiovascular risk profile in the setting of numerous established comorbidities.

Left ventricular thrombus formation following myocardial infarction reflects the principles of Virchow’s triad, including endocardial injury, blood stasis from impaired ventricular contraction, and hypercoagulability. Established predictors include anterior myocardial infarction, reduced LVEF (<40%), LV aneurysm formation, severe apical akinesis, and larger infarct size [11-13]. Our patient’s proximal left anterior descending artery disease and severe LV dysfunction with LVEF of 23% represent major risk factors for thrombus formation. Although nephrotic-range proteinuria associated with diabetic kidney disease may also contribute to hypercoagulability, her severe ischemic cardiomyopathy alone plausibly explains the development of LV thrombus.

Embolic stroke occurs in approximately 10-12% of patients with LV thrombus after myocardial infarction, with the highest risk occurring within the first month. LV thrombus confers an estimated 5.5-fold increased risk of systemic embolization compared with patients without thrombus. Mobile and protruding thrombi are associated with particularly high embolic risk. Cardiac MRI demonstrates superior sensitivity compared with standard transthoracic echocardiography for thrombus detection, and serial imaging is recommended to assess thrombus persistence and guide duration of anticoagulation therapy [13-15].

Decisions regarding revascularization required individualized multidisciplinary assessment. CABG is generally recommended in patients with multivessel disease involving the proximal left anterior descending artery, particularly in the setting of diabetes mellitus or LV dysfunction. Young patients with complex multivessel CAD derive significant long-term benefit from CABG compared with PCI. However, recent stroke and dialysis dependence substantially increase perioperative risk [16-18]. Initial surgical deferral in this case, therefore, reflected appropriate risk stratification and optimization prior to eventual successful revascularization.

This case highlights the intersection of premature atherosclerosis, advanced ischemic cardiomyopathy, thromboembolic complications, and progressive renal disease in a young adult with longstanding poorly controlled metabolic risk factors.

## Conclusions

This case is notable for the patient’s exceptionally young age and the convergence of advanced cardiovascular and systemic complications, including severe multivessel CAD requiring CABG, profound ischemic cardiomyopathy with LV thrombus, embolic stroke despite therapeutic anticoagulation, and progression to dialysis-dependent end-stage renal disease. Such a constellation of disease is rare in a 31-year-old patient and underscores the aggressive trajectory that longstanding poorly controlled metabolic disease can produce.

Clinicians should maintain a high index of suspicion for premature CAD and LV thrombus in young patients with multiple cardiovascular risk factors presenting with heart failure symptoms or elevated cardiac biomarkers. Management requires careful coordination of antithrombotic therapy, heart failure optimization, renal replacement considerations, neurologic risk stratification, and timing of surgical revascularization. Early multidisciplinary collaboration is essential to balance thromboembolic risk, bleeding risk, operative timing, and renal complications in these highly complex presentations of premature ischemic heart disease.
